# Dr. George Washington Matteson

**Published:** 1910-05

**Authors:** 


Dr. George Washington Matteson, of Middleville, Mich., died at his home February 24, 1910, of senile debility, aged 88 years.

He graduated from the University of Buffalo, Medical Department, February 23, 1855.

